# Excretory/secretory products of *Angiostrongylus cantonensis* fifth-stage larvae induce endoplasmic reticulum stress *via* the Sonic hedgehog pathway in mouse astrocytes

**DOI:** 10.1186/s13071-020-04189-w

**Published:** 2020-06-18

**Authors:** Kuang-Yao Chen, Yi-Ju Chen, Chien-Ju Cheng, Kai-Yuan Jhan, Lian-Chen Wang

**Affiliations:** 1 Department of Parasitology, School of Medicine, China Medical University, Taichung, 404 Taiwan; 2 Department of Parasitology, College of Medicine, Chang Gung University, Taoyuan, 333 Taiwan; 3 Graduate Institute of Biomedical Sciences, College of Medicine, Chang Gung University, Taoyuan, 333 Taiwan; 4 Molecular Infectious Disease Research Center, Chang Gung Memorial Hospital, Taoyuan, Taiwan

**Keywords:** *Angiostrongylus cantonensis*, Fifth-stage larvae, Excretory/secretory products, Astrocytes, Endoplasmic reticulum stress, Sonic hedgehog pathway

## Abstract

**Background:**

*Angiostrongylus cantonensis* is an important food-borne zoonotic parasite. Humans are non-permissive hosts, and this parasite develops into fifth-stage larvae (L5) in the brain and subarachnoid cavity and then induces eosinophilic meningitis and eosinophilic meningoencephalitis. Excretory/secretory products (ESPs) are valuable targets for the investigation of host-parasite interactions. These products contain a wide range of molecules for penetrating defensive barriers and avoiding the immune response of the host. Endoplasmic reticulum (ER) stress has been found to be associated with a wide range of parasitic infections and inflammation. ER stress can increase cell survival *via* the activation of downstream signalling. However, the mechanisms of ER stress in *A. cantonensis* infection have not yet been clarified. This study was designed to investigate the molecular mechanisms of ER stress in astrocytes after treatment with the ESPs of *A. cantonensis* L5.

**Results:**

The results demonstrated that *A. cantonensis* infection activated astrocytes in the mouse hippocampus and induced the expression of ER stress-related molecules. Next, the data showed that the expression of ER stress-related molecules and the Ca^2+^ concentration were significantly increased in activated astrocytes after treatment with the ESPs of L5 of *A. cantonensis*. Ultimately, we found that ESPs induced GRP78 expression *via* the Sonic hedgehog (Shh) signalling pathway.

**Conclusions:**

These findings suggest that in astrocytes, the ESPs of *A. cantonensis* L5 induce ER stress and that the Shh signalling pathway plays an important role in this process.
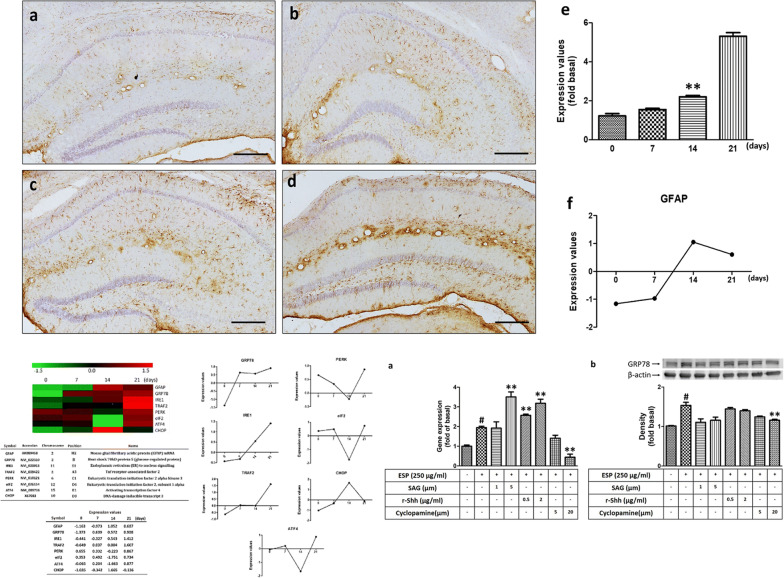

## Background

*Angiostrongylus cantonensis* is an important causative agent of human cerebral angiostrongyliasis, such as eosinophilic meningitis and eosinophilic meningoencephalitis. During infection, fifth-stage larvae (L5) can induce a wide range of inflammatory responses, including eosinophil recruitment and cytokine secretion in the brains of humans [1, 2]. The clinical manifestations include headache, fever, nausea, vomiting, neck stiffness and paraesthesia. This disease is considered to be an endemic disease in Southeast Asia and Pacific islands [3, 4]. Recently, human cerebral angiostrongyliasis has become an emerging disease in many parts of the world, including China, Taiwan, Thailand, the USA, including Hawaii, Brazil and the Caribbean islands, including Jamaica [5–13]. Moreover, infections have been recently reported in more than 30 countries [4]. Infection in humans is acquired by ingesting infective third-stage larvae (L3) of *A. cantonensis* in intermediate hosts or paratenic hosts, such as snails, slugs, frogs, fish, freshwater crustaceans and vegetables [14].

The endoplasmic reticulum (ER) is an organelle that has multiple complex functions, including protein synthesis, cellular calcium (Ca^2+^) storage, lipid biosynthesis, and membrane biogenesis [15, 16]. The generation of ER stress due to the accumulation of unfolded and misfolded proteins in the ER may activate the unfolded protein response (UPR) and then induce the activation of related signalling pathways. When this stress can be alleviated, the UPR can lead to cell apoptosis [17]. First, ER stress activates an important molecule, glucose-regulated protein 78 (GRP78). GRP78, also called binding immunoglobulin protein (BiP), is an ER stress marker and chaperone in the heat shock protein family [18, 19]. Under ER stress, the activation of GRP78 may increase cell survival through the UPR [20]. In addition, the induction of GRP78 may also protect cells from ER stress-induced apoptosis by activating Bcl-2 and inhibiting Bak, Bax and caspase [21, 22].

In mammalian cells, GRP78 activates three signalling pathways in parallel through transmembrane ER stress sensors (IRE1, PERK and ATF6) [23, 24]. IRE1 can induce the splicing of the cytoplasmic transcription factor XBP1 to spliced XBP1 (XBP1s) and activate the gene expression of chaperones, autophagy, and inflammation. Activated PERK phosphorylates eIF2α to reduce the load of unfolded proteins by attenuating translation. In addition, the transcription factor ATF4 can stimulate autophagy by inducing CHOP expression. On the other hand, ATF6 translocates to the Golgi and reduces protein accumulation by upregulating XBP1 expression.

Hh has three homologs, namely, Sonic hedgehog (Shh), Desert hedgehog (Dhh) and Indian hedgehog (Ihh), but only Shh is broadly expressed in different tissues [25]. Sonic hedgehog (Shh) signalling plays an important role in animal development. Shh signalling can trigger other common signalling pathways. When Shh is activated and secreted, this protein can interact with the transmembrane protein Patched (Ptch). Under these conditions, Smoothened (Smo) and the transcription factor Glioma-associated oncogene-1 (Gli) can be activated [26–28].

In our previous studies, we found that *A. cantonensis* infection in mice may enhance the expression of GRP78 and that the activation of the Shh signalling pathway can reduce cell death *via* the GRP78-dependent pathway [29]. On the other hand, oxidative stress and cell apoptosis can be induced in astrocytes after treatment with the excretory/secretory products (ESPs) of *A. cantonensis* fifth-stage larvae (L5). However, ROS (superoxide and hydrogen superoxide) and the apoptosis of astrocytes are decreased after Shh signalling pathway activation, and the activity of antioxidants is elevated after ESP treatment [30]. Therefore, we demonstrated that the ESPs of *A. cantonensis* L5 can induce oxidative stress and cell apoptosis and that the Shh signalling plays an important role in the protection of astrocytes. In the present study, we investigated the molecular mechanisms of ER stress in mouse brain astrocytes after treatment with the ESPs of *A. cantonensis* L5. The results suggested that the ESPs of *A. cantonensis* L5 induce ER stress in astrocytes and that the activation of the Shh signalling pathway can stimulate GRP78 expression.

## Methods

### Parasite and animals

*Angiostrongylus cantonensis* (Taipei strain) has been maintained in our laboratory in Sprague-Dawley (SD) rats and *Biomphalaria glabrata* snails since 1980 [30, 31]. SD rats and BALB/c mice were purchased from the National Laboratory Animal Center (Taipei, Taiwan) and BioLASCO Taiwan Co., Ltd. (Taipei, Taiwan). Third-stage larvae (L3) of *A. cantonensis* were collected from infected *Biomphalaria glabrata* by digestion with 0.6% (w/v) pepsin-HCl (pH 2–3) at 37 °C for 45 min on day 21 post-infection [32]. Each rat or BALB/c mouse was inoculated with L3 *via* stomach intubation. Rats and mice were kept in plastic cages and provided with food and water *ad libitum*. The experimental animals were sacrificed by anaesthesia with isoflurane (1 ml/min) (Panion & BF Biotech Inc., Taipei, Taiwan).

### Preparation of ESPs of *A. cantonensis*

Live fifth-stage larvae (L5) of *A. cantonensis* were isolated from the brain tissues of infected rats by anaesthetisation with isoflurane 21 days post-infection. After the worms were washed with saline, phosphate-buffered saline (PBS), distilled water and RPMI containing a high concentration of antibiotic antimycotic solution (Sigma-Aldrich, St. Louis, USA), they were incubated in RPMI without fetal bovine serum (FBS) for 24, 48 and 72 h. The excretory/secretory products (ESPs) of *A. cantonensis* L5 were collected from the culture medium and concentrated with Amicon Ultra-15 10K centrifugal filter devices (Merck Millipore, Burlington, USA). The concentration of ESPs was detected by a Bio-Rad Protein Assay Kit (Bio-Rad, Hercules, USA) according to the manufacturer’s instructions. These concentrated ESPs were utilized to treat mouse astrocytes, and mRNA and protein expression levels were detected in astrocytes [33].

### Astrocyte culture

Astrocytes from the mouse brain (CRL-2535) were obtained from the American Type Culture Collection (ATCC) and employed in this research. The cells were cultured in Dulbecco’s modified Eagle’s medium (Corning, Corning, USA) supplemented with 10% foetal bovine serum and 100 U/ml penicillin/streptomycin in poly-L-lysine-coated culture flasks at 37 °C under 5% CO_2_. Finally, the cells were pre-treated with recombinant Shh (r-Shh), Shh agonist (SAG), and cyclopamine (Cyclo) for 1 h and then incubated with 250 μg/ml excretory/secretory products (ESPs) of *A. cantonensis* L5 for 12 h [29].

### Brain specimen collection, immunohistochemistry and immunofluorescence staining

After mice were completely anaesthetised by inhalation of 3% (v/v) isoflurane, potassium phosphate-buffered saline (KPBS) was perfused through the heart. The mouse brains were collected from the cranial cavities and then immediately mounted and stored in optimal cutting temperature (OCT) medium (Sakura Finetek, Flemingweg, Netherlands) for further experiments. Before staining, the frozen tissue sections were fixed in 2% (w/v) PFA (paraformaldehyde) and permeabilized in 0.5% (v/v) Triton X-100. The sections were immersed in 5% (v/v) goat serum for 1 h and placed in primary antibody (anti-GFAP) (Abcam, Cambridge, UK) at 4 °C overnight. The sections were placed in secondary antibodies at room temperature for 1 h. avidin-biotin-peroxidase complex reagent (Vector Laboratories, Inc., Burlingame, USA) and DAB (3.3’-diaminobenzidine) reaction solution were added to each section. Finally, the sections were examined by light microscopy.

### RNA extraction and cDNA microarray analysis

Total RNA was extracted from astrocytes (in 10 cm culture dishes) treated with the indicated doses of ESPs of *A. cantonensis* L5 by using the GENEzol TriRNA Pure Kit (Geneaid, New Taipei, Taiwan). The concentration of RNA was determined with a spectrophotometer (OD260 nm). The cDNA targets for hybridization were synthesized by reverse transcription of each RNA sample in the presence of Cy5-dUTP and Cy3-dUTP (Amersham Pharmacia Biotech, Amersham, UK). A customized *A. cantonensis* cDNA microarray (version 2.0) was utilized, and the data were analysed by QuantArray software (GSI Lumonics, Rugby, UK).

### Real-time qPCR

First-strand cDNA was synthesized using the iScript™ Advanced cDNA Synthesis Kit (Bio-Rad) with random hexamers according to the manufacturer’s instructions. Real-time qPCR was performed using iQ™ SYBR® Green Supermix (Bio-Rad) on the CFX Connect™ Real-Time PCR Detection System (Bio-Rad). GAPDH was used as the internal control. Expression levels were detected with specific primers (Table 1).

**Table 1:** Primer sequences for real-time qPCR

Gene		Sequence (5’-3’)
*Gfap*	Forward	CAGATCCGAGAAACCAGCCT
	Reverse	GAGCCTGGCAAACAGGACTA
*Grp78*	Forward	GTGTGTGAGACCAGAACCGT
	Reverse	AACACACCGACGCAGGAATA
*Perk*	Forward	TTTCCATCCTCAGCCCCACA
	Reverse	GGCACTCACGGAGTCGTATT
*eif2α*	Forward	TTACTGTACGCCTGCGCTTT
	Reverse	CTTCTCACAGCACCGCACTA
*Atf4*	Forward	CGGCTGGTCGTCAACCTATAA
	Reverse	GGGGTAACTGTGGCGTTAGA
*Chop*	Forward	GAGCCAGAATAACAGCCGGA
	Reverse	TCTGCTTTCAGGTGTGGTGG
*Ire1*	Forward	CCCGGGAAATACATGAGCCA
	Reverse	CCAGCGGAGGACAAGGAAAT
*Traf2*	Forward	AAGTACCTCTGTTCAGCCTGC
	Reverse	AGAGAATGGATGCACACCTGA
*Atf6*	Forward	GGGAATGGAAGCCTAAAGAGGA
	Reverse	ACAGAGAAACAAGCTCGGTGT
*Gapdh*	Forward	GGTCCCAGCTTAGGTTCATCA
	Reverse	TTTGCCGTGAGTGGAGTCAT

### SDS-PAGE electrophoresis and western blotting analysis

Total protein extracted from astrocytes was separated by 12% SDS-PAGE. The separated proteins were transferred to a nitrocellulose (NC) membrane and incubated with antibodies against GFAP (Proteintech, Rosemont, USA), Shh (Abcam), Ptch (Sigma-Aldrich), Smo (Sigma-Aldrich), Gli-1 (Sigma-Aldrich), GRP78 (Proteintech), PERK, eIF2 (Proteintech), phospho-eIF2 (EnoGene Biotech Co., New York, USA), IRE1 (Signalway Antibody, Baltimore, USA), phospho-IRE1 (Boster, Pleasanton, USA), CHOP (Proteintech), and β-actin (Proteintech). The NC membrane was washed with TBS/T three times and then incubated with a 1:10,000 dilution of anti-rabbit or mouse horseradish peroxidase antibody (Sigma-Aldrich). The bands were detected by ECL reagents (EMD Millipore, Burlington, USA) and captured by a ChemiDoc Imaging System (Bio-Rad). ImageJ software analysis was employed to detect the optical density of the target proteins.

### Ca^2+^ analysis

The concentration of Ca^2+^ was measured using the Calcium Detection Assay Kit (Abcam). The samples were pre-treated with the ESPs of *A. cantonensis* L5 and then treated with Chromogenic Reagent and Calcium Assay Buffer at room temperature for 10 min protected from light. After incubation, the samples and standards were analysed with a spectrophotometer (OD575 nm).

### Statistical analysis

Student’s t-test was employed to compare the mRNA and protein expression levels using GraphPad Prism 5 software (GraphPad Software, San Diego, CA, USA). The data are expressed as the mean ± standard deviation. *P* < 0.05 and < 0.01 were considered statistically significant.

## Results

### The activation of astrocytes in mouse brains after *A. cantonensis* infection

**Fig. 1:**
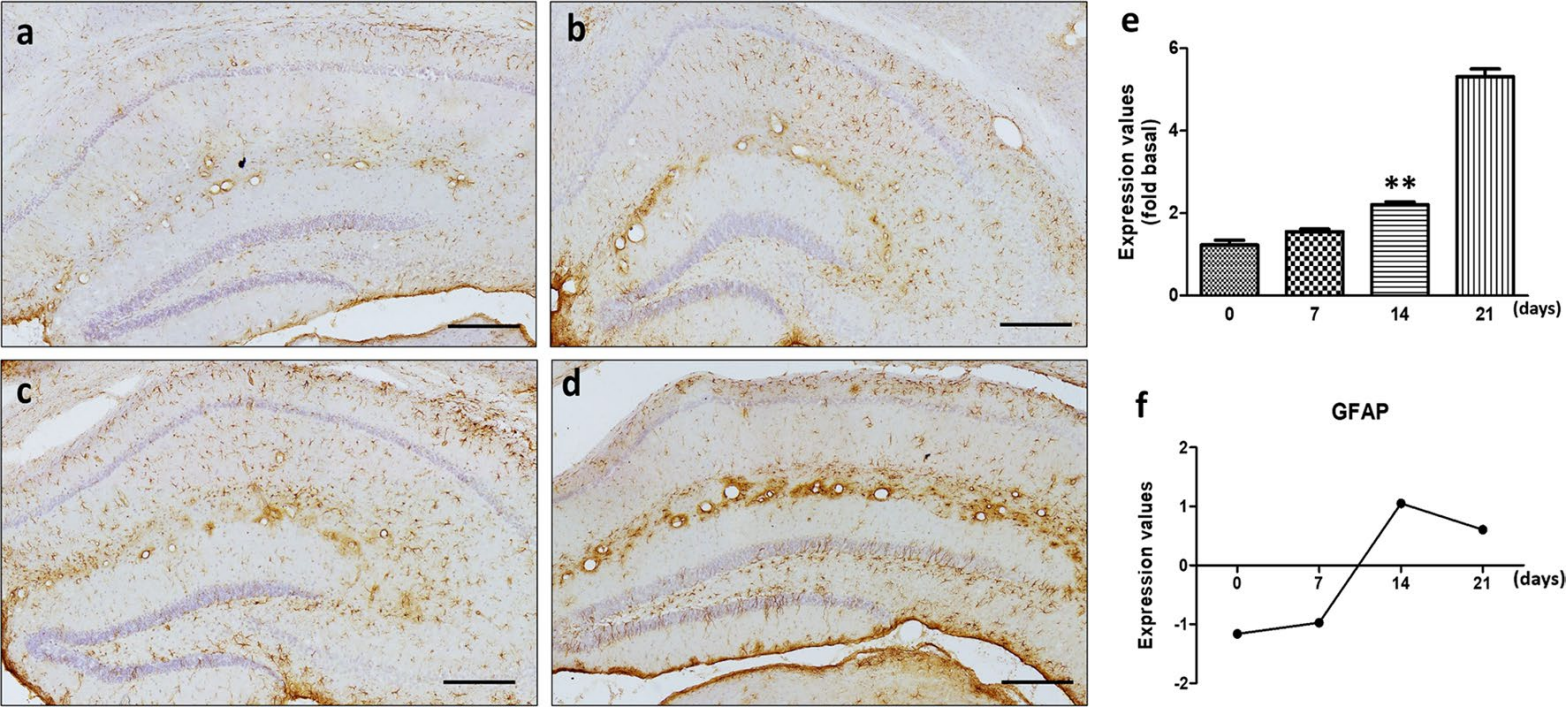
Astrocyte activation was induced in the brains of *Angiostrongylus cantonensis-*infected mice. The expression of GFAP was detected in the hippocampus in the absence of infection (**a**) and on days 7 (**b**), 14 (**c**) and 21 (**d**) post-infection after infection with 25 third-stage larvae. **e** The expression level of GFAP in the brain was quantified by ImageJ software (**P < 0.01). **f** The mRNA expression level of GFAP in the brain was detected by microarray analysis

Astrocytes are the most abundant glial cells in the central nervous system (CNS). These cells can regulate the migration and differentiation of neural stem cells or other glial cells by secreting factors [34, 35]. Astrocytes can also reduce neuronal death during oxidative stress [36]. Glial fibrillary acidic protein (GFAP), an intermediate filament protein, is highly expressed in activated astrocytes [37–39]. GFAP is the most commonly used cell-specific marker for astrocytes in neurological studies. This marker can be employed to distinguish activated astrocytes from other brain cells. In this study, we used immunohistochemical (IHC) and cDNA microarray analysis to detect the expression of GFAP in mouse brains after *A. cantonensis* infection. IHC staining with an anti-GFAP antibody revealed that the expression of GFAP in astrocytes was significantly higher around the hippocampus after day 21 post-infection (*t*_(4)_ = 7.244, *P* < 0.01) (Fig. 1a–e). On the other hand, the mRNA expression of GFAP in mouse brains was elevated after *A. cantonensis* infection (Fig. 1f). These data indicated that astrocytes were activated in the brain after *A. cantonensis* infection.

### The expression of ER stress-related genes in the mouse brain after *A. cantonensis* infection

**Fig. 2:**
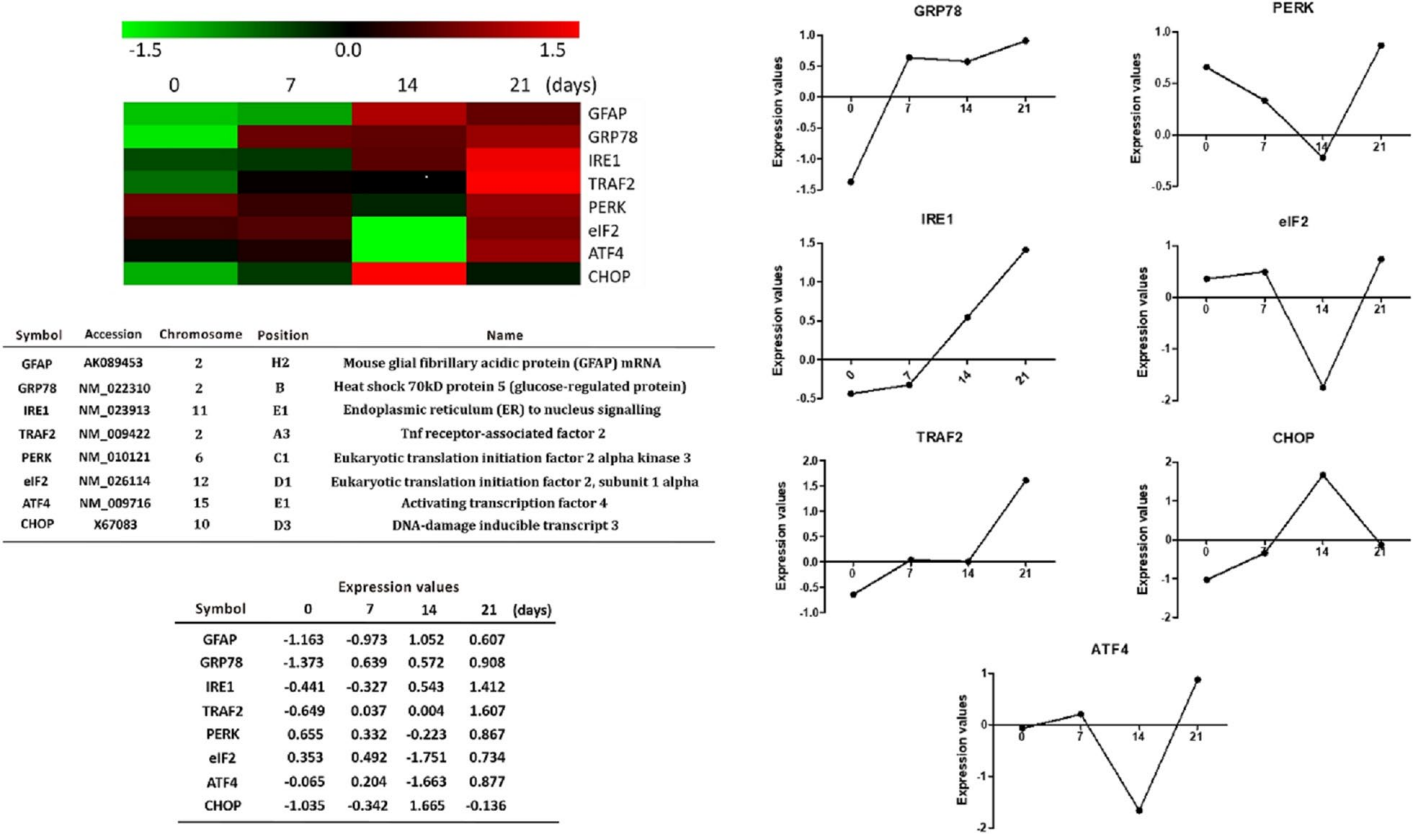
*Angiostrongylus cantonensis* infection stimulates the expression of ER stress-related molecules in the mouse brain. Mouse brains were collected from *A. cantonensis*-infected mice on days 0, 7, 14 and 21 post-infection. Then, the mRNA expression levels of ER stress-related molecules were detected by cDNA microarray analysis

To evaluate the induction of ER stress in the mouse brain after *A. cantonensis* infection, we collected 25 third-stage larvae of *A. cantonensis* to infect BALB/c mice. cDNA microarray analysis was utilized to detect the mRNA expression of ER stress-related genes in four mouse brains (day 0, 7, 14 and 21 post-infection), including *GRP78*, *IRE1*, *TRAF2*, *PERK*, *eIF2*, *ATF4* and *CHOP*. The data indicated a trend of elevated mRNA expression levels (Fig. 2). These results suggest that ER stress is induced in the mouse brain after *A. cantonensis* infection.

### ESPs induce astrocyte activation and GRP78 expression

**Fig. 3:**
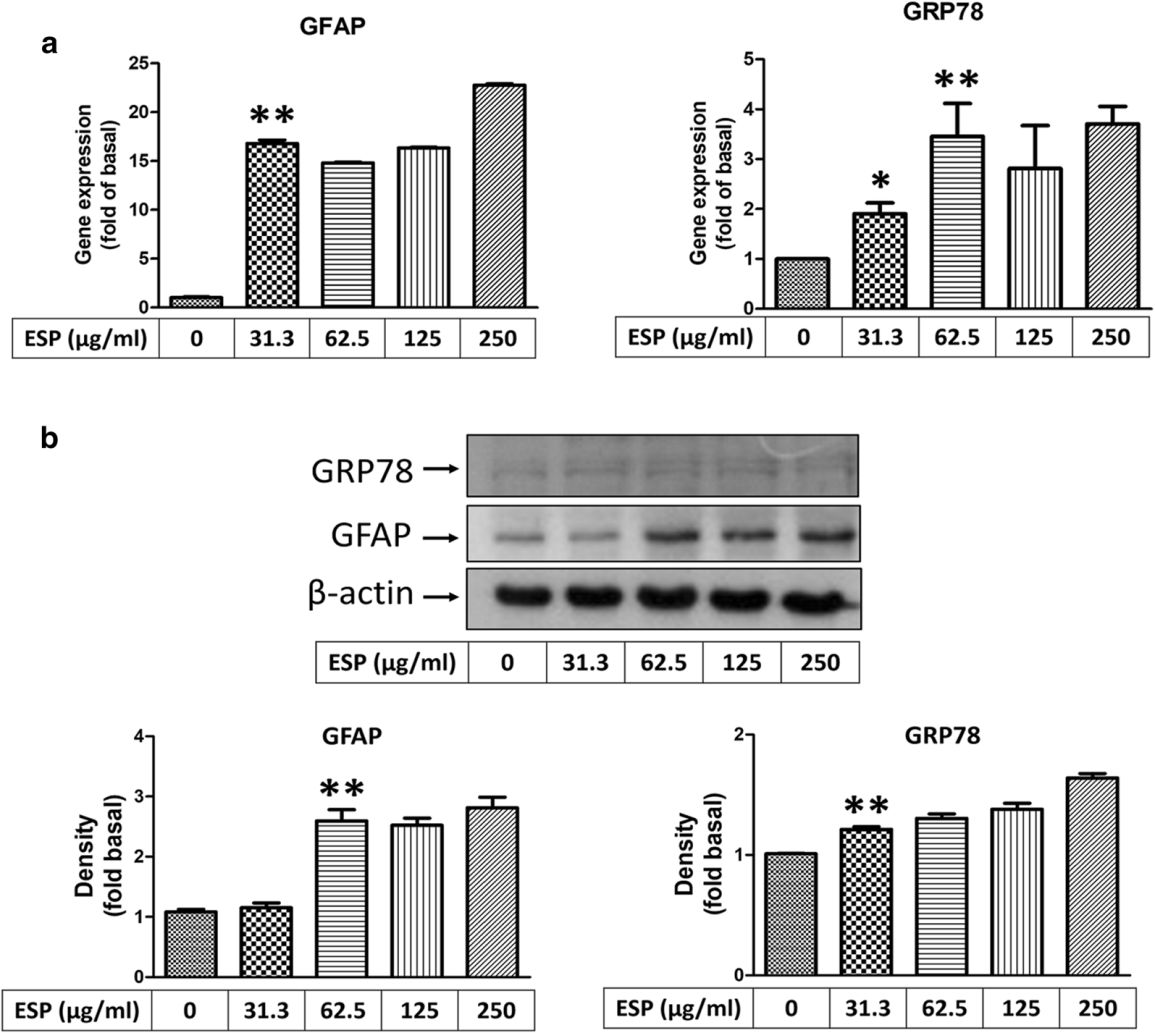
Excretory/secretory products of *Angiostrongylus cantonensis* L5 stimulate astrocyte activation and GRP78 expression. Cells were treated with 0, 31.3, 62.5, 125 or 250 μg/ml excretory/secretory products (ESPs) of *A. cantonensis* L5 for 12 h. The mRNA (**a**) and protein (**b**) expression levels of GFAP and GRP78 were detected by real-time qPCR and western blotting. The data are expressed as the mean ± SD from three independent experiments (*n* = 3). *P < 0.05 and **P < 0.01 compared with cells treated with 0 μg/ml ESPs of *A. cantonensis* L5 for 12 h

To investigate the activation of astrocytes and GRP78 expression, cells were treated with ESPs for 12 h. Real-time qPCR and western blotting were used to monitor the mRNA and protein expression levels of GFAP (mRNA level: *t*_(4)_ = 41.44, *P* < 0.0001; protein level: *t*_(4)_ = 7.892, *P* < 0.01) and GRP78 (mRNA level: *t*_(4)_ = 4.814, *P* < 0.01; protein level: *t*_(4)_ = 8.993, *P* < 0.001). The results showed that the ESPs of *A. cantonensis* stimulated the expression of GFAP and GRP78 in a dose-dependent manner (Fig. 3a, b).

### ESPs induce the expression of ER stress-related molecules in astrocytes

**Fig. 4:**
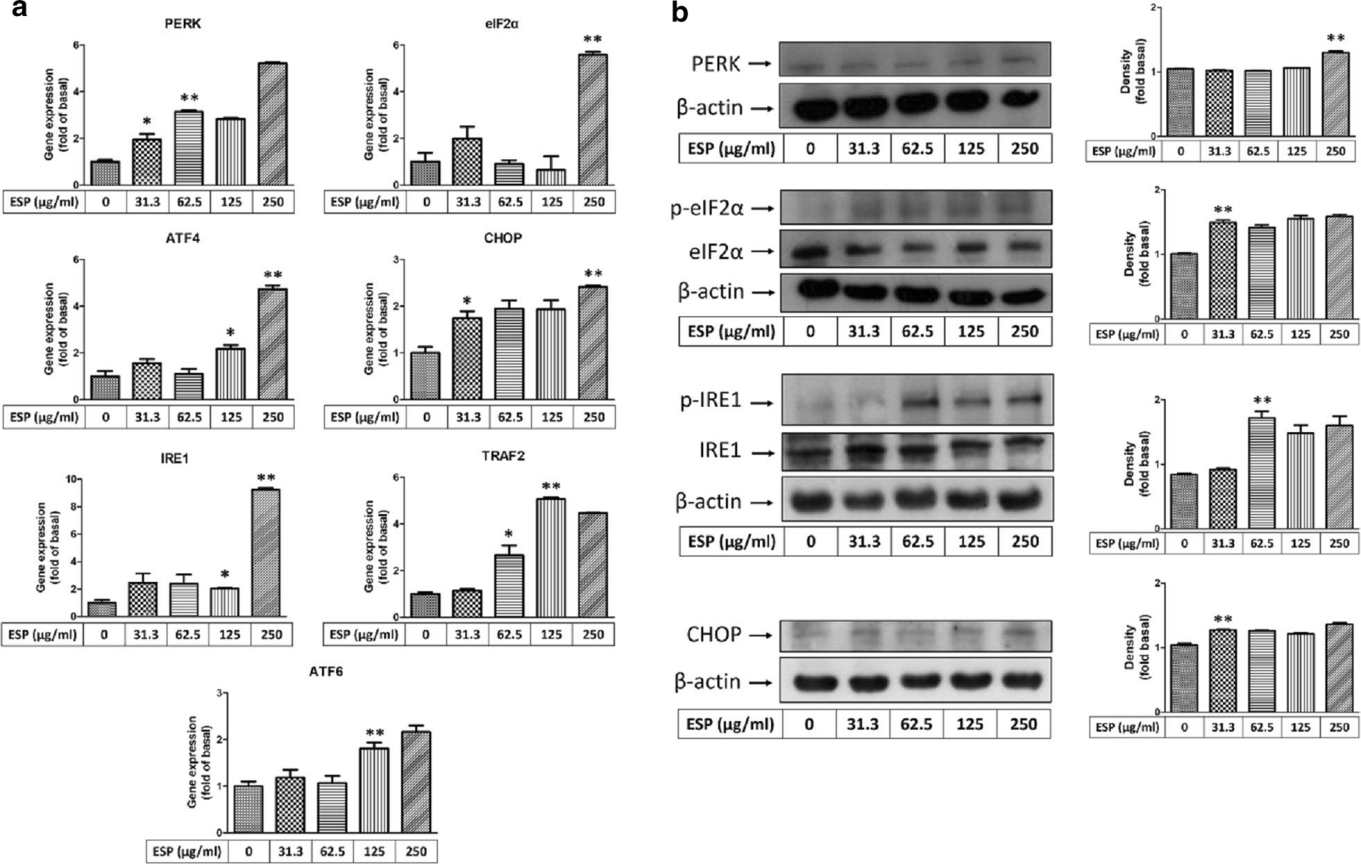
Excretory/secretory products of *Angiostrongylus cantonensis* L5 stimulate the activation of ER stress-related pathways in astrocytes. Cells were treated with 0, 31.3, 62.5, 125 or 250 μg/ml excretory/secretory products (ESPs) of *A. cantonensis* L5 for 12 h. The mRNA (**a**) and protein (**b**) expression levels of ER stress-related molecules were detected by real-time qPCR and western blotting. The data are expressed as the mean ± SD from three independent experiments (*n* = 3). *P < 0.05 and **P < 0.01 compared cells treated with 0 μg/ml ESPs of *A. cantonensis* L5 for 12 h

As shown in Fig. 3, the results demonstrated that the ESPs of *A. cantonensis* induced astrocyte activation and GRP78 expression. Thus, we further examined whether the ESPs of *A. cantonensis* activated the ER stress downstream pathway. We detected the expression of ER stress-related genes and proteins (PERK, eIF2, ATF4, CHOP, IRE1, TRAF2 and ATF6) in astrocytes after treatment with ESPs for 12 h. The results of real-time qPCR and western blot analysis showed that the expression levels of ER stress-related molecules in astrocytes were increased in a dose-dependent manner (PERK: mRNA level: *t*_(4)_ = 20.48, *P* < 0.0001; protein level: *t*_(4)_ = 9.671, *P* < 0.001; eIF2: mRNA level: *t*_(4)_ = 11.51, *P* < 0.001; protein level: *t*_(4)_ = 11.92, *P* < 0.001; ATF4: *t*_(4)_ = 13.41, *P* < 0.001; CHOP: mRNA level: *t*_(4)_ = 10.70, *P* < 0.001; protein level: *t*_(4)_ = 8.752, *P* < 0.001; IRE1: mRNA level: *t*_(4)_ = 32.51, *P* < 0.0001; protein level: *t*_(4)_ = 8.797, *P* < 0.001; TRAF2: *t*_(4)_ = 38.43, *P* < 0.0001; ATF6: *t*_(4)_ = 4.841, *P* < 0.01) (Fig. 4a, b). These data indicated that the ESPs of *A. cantonensis* induce the elevation of ER stress.

### ESPs induce Ca^2+^ release in astrocytes

**Fig. 5:**
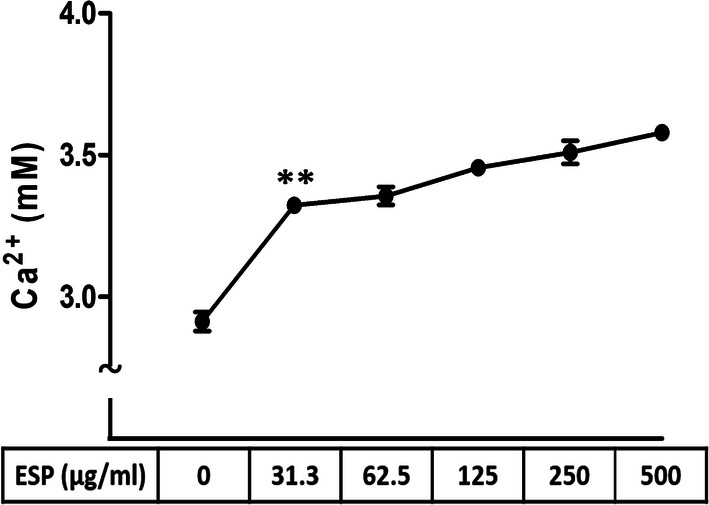
Excretory/secretory products of *Angiostrongylus cantonensis* L5 stimulate calcium secretion in astrocytes. Cells were treated with 0, 31.3, 62.5, 125 or 250 μg/ml excretory/secretory products (ESPs) of *A. cantonensis* L5 for 12 h. The concentration of calcium was measured by the Calcium Assay Kit. The data are expressed as the mean ± SD from three independent experiments (*n* = 3). **P < 0.01 compared with cells treated with 0 μg/ml ESPs of *A. cantonensis* L5 for 12 h

Some previous studies demonstrated that a loss of Ca^2+^ cellular homeostasis can induce ER stress and ER stress-related apoptosis [40–42]. To examine whether the ESPs of *A. cantonensis* induce the elevation of the Ca^2+^ concentration in astrocytes, we used the calcium detection assay kit to detect the concentration of Ca^2+^ in different dose of ESPs treatment (0, 31.3, 62.5, 125 or 250 μg/ml). The results showed that the concentration of Ca^2+^ was increased in astrocytes in a dose-dependent manner (2.92 to 3.58 mM) (*t*_(4)_ = 11.39, *P* < 0.001) (Fig. 5).

### ESPs induce the expression of GRP78 *via* the Shh signalling pathway

**Fig. 6:**
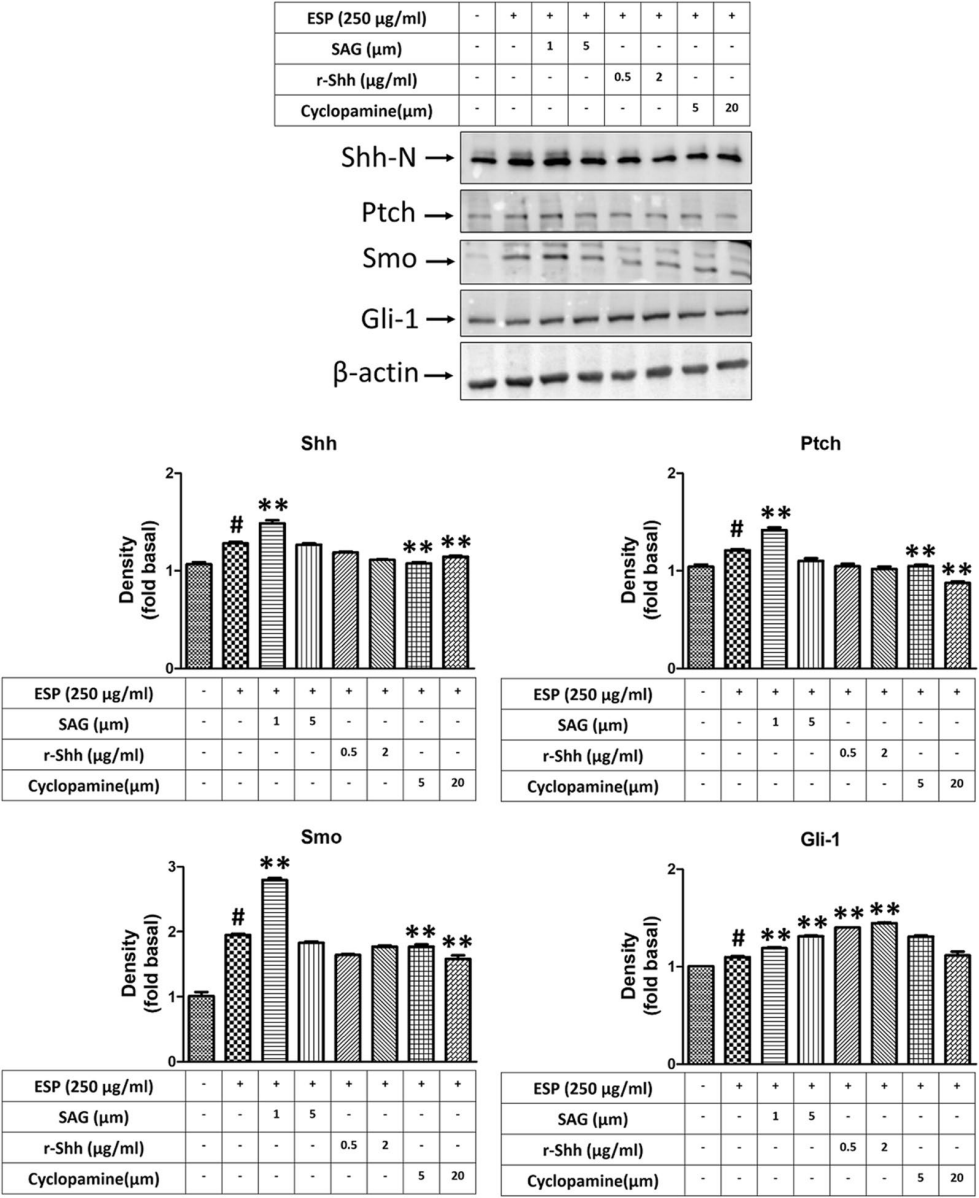
Evaluation of the effectiveness of an activator or inhibitor on the Shh signalling pathway. Cells were pre-treated with recombinant Shh (r-Shh), Shh agonist (SAG) and cyclopamine (Cyclo) for 1 h, then incubated with 250 μg/ml excretory/secretory products (ESPs) of *A. cantonensis* L5 for 12 h. The protein expression levels were detected by western blotting. The data are expressed as the mean ± SD from three independent experiments (*n* = 3). ^#^*P <* 0.01 compared with the control; **P < 0.01 compared with cells exposed to ESPs

**Fig. 7:**
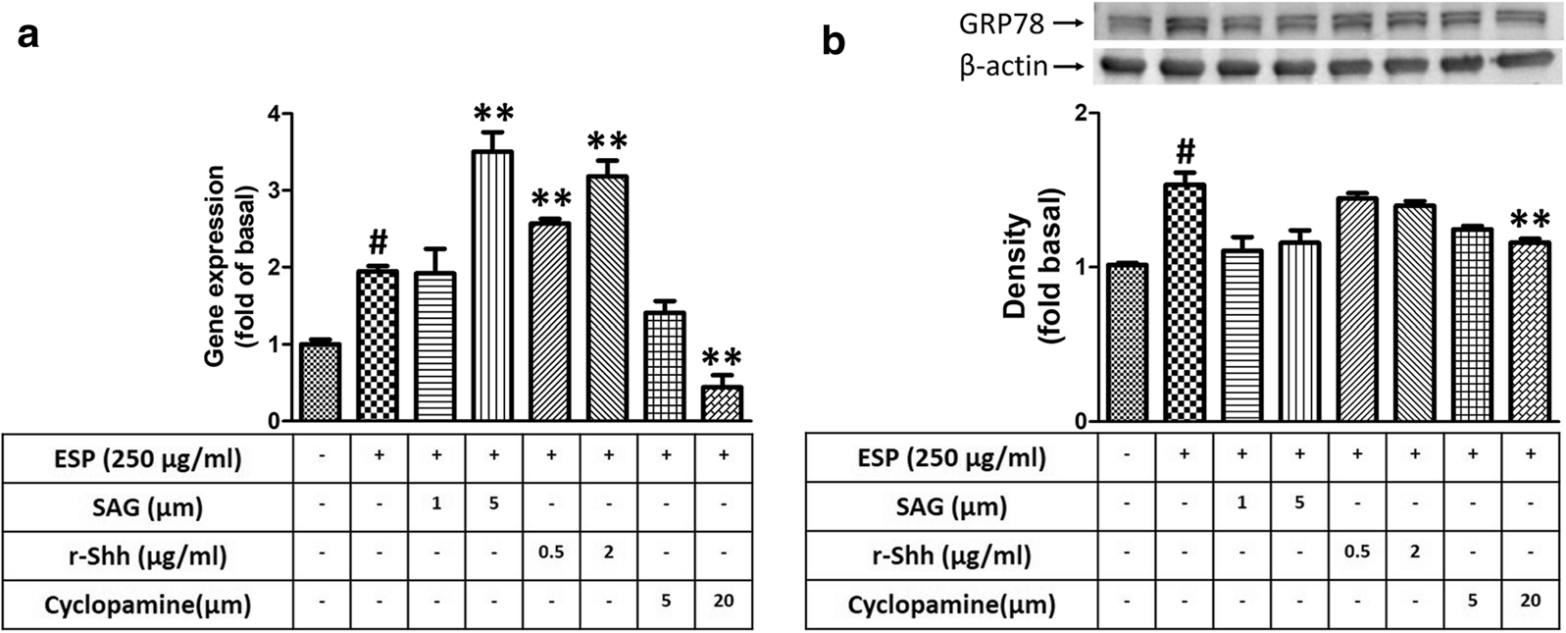
Excretory/secretory products induce GRP78 expression through the Shh signalling pathway. Cells were pre-treated with recombinant Shh (r-Shh), Shh agonist (SAG) and cyclopamine (Cyclo) for 1 h, then incubated with 250 μg/ml excretory/secretory products (ESPs) of *A. cantonensis* L5 for 12 h. The mRNA (**a**) and protein (**b**) expression levels were detected by real-time qPCR and western blotting. The data are expressed as the means ± SD from three independent experiments (*n* = 3). ^#^*P <* 0.01 compared with the control; **P < 0.01 compared with cells exposed to ESPs

To determine whether Shh signalling can induce ER stress generation in astrocytes after treatment with the ESPs of *A. cantonensis*, cells were pre-treated with a Smo agonist (SAG), recombinant Shh (r-Shh), and an Shh pathway inhibitor (cyclopamine) and then treated with ESPs. First, we wanted to evaluate the effectiveness of an activator and inhibitor on the Shh signalling pathway. Western blotting analysis was used to confirm the protein expression of Shh pathway-related molecules, including Shh-N, Ptch, Smo, and Gli-1. The data showed that the expression of Shh pathway-related proteins was elevated in astrocytes after SAG (Shh-N: *t*_(4)_ = 5.908, *P* < 0.01; Ptch: *t*_(4)_ = 7.054, *P* < 0.01; Smo: *t*_(4)_ = 23.42, *P* < 0.0001; Gli-1: *t*_(4)_ =16.95, *P* < 0.0001) and r-Shh treatment (Gli-1: *t*_(4)_ = 26.93, *P* < 0.0001). Conversely, the expression of Shh pathway-related proteins was decreased by cyclopamine treatment (Shh-N: *t*_(4)_ = 9.347, *P* < 0.001; Ptch: *t*_(4)_ = 18.12, *P* < 0.0001; Smo: *t*_(4)_ = 6.351, *P* < 0.01) (Fig. 6). Next, real-time qPCR and western blotting analysis were employed to detect the expression of GRP78 in astrocytes after treatment with the ESPs of *A. cantonensis*. The results showed that the expression level of GRP78 was significantly changed following Shh pathway activation (SAG: *t*_(4)_ = 5.989, *P* < 0.01; r-Shh: *t*_(4)_ = 6.600, *P* < 0.01) or inactivation (mRNA level: *t*_(4)_ = 8.710, *P* < 0.001; protein level: *t*_(4)_ = 4.743, *P* < 0.01) after ESPs treatment (Fig. 7). These data indicate that the ESPs of *A. cantonensis* L5 induced ER stress in astrocytes through the Shh signalling pathway.

## Discussion

In the life-cycle of *A. cantonensis*, the first-stage larvae (L1) are released into the faeces of the definitive host (rat). L1 in the faeces can infect the intermediate host and become third-stage larvae (L3). When humans are infected with *A. cantonensis* by eating L3 in intermediate hosts or paratenic hosts, infective L3 can penetrate and migrate into the central nervous system (CNS) through the circulatory system. Afterwards, L3 develop into fifth-stage larvae (L5) and remain in the CNS of the host indefinitely. In this condition, eosinophilic meningitis and eosinophilic meningoencephalitis can be induced in the brain of the host [33]. In our histopathological study, we found that L5 were surrounded by eosinophils in the anterior cerebral fissure, hippocampus, posterior cerebral fissure, and cerebellar fissure on day 14 post-infection [43]. On the other hand, *A. cantonensis* infection can induce a wide range of pathological changes in the CNS, including infiltration of eosinophils and congestion in the meninges, infiltration of lymphocytes and eosinophils in the meninges and choroid plexus, and necrosis and perivascular cuffing of lymphocytes in the brain parenchyma [44]. In this study, we also demonstrated that astrocytes were significantly activated in the hippocampus after *A. cantonensis* infection.

In the present study, the experiments were performed to determine the influence of the ESPs of *A. cantonensis* L5 in mouse astrocytes. The excretory/secretory products (ESPs) of nematodes, trematodes and cestodes contain a wide range of molecules, including proteins, lipids, glycans, and nucleic acids, and they can aid in the penetration of host defensive barriers, the avoidance of the host immune response and establishment and survival in host tissues [45–49]. ESPs are also useful targets for investigating the interaction between parasitic helminths and hosts [50–52]. In studies on the ESPs of *A. cantonensis*, proteomic analysis has been employed to determine the composition of ESPs in adults. Aspartyl protease inhibitor, cathepsin B-like cysteine proteinase, haemoglobinase- type cysteine proteinase, and heat shock protein 70 have been detected in ESPs [53]. On the other hand, our study identified approximately 51 protein spots and found immunoreactivity for protein disulfide-isomerase, a putative aspartic protease, and annexin in *A. cantonensis* L5 [33]. Here, we found that the ESPs of *A. cantonensis* L5 can stimulate the ER stress generation and the Ca^2+^ concentration in activated astrocytes.

ER stress has been found to be associated with a wide range of parasitic infections, including *Trichinella spiralis*, *Toxoplasma gondii*, *Trypanosoma brucei* and *Plasmodium falciparum*, and inflammation [54–58]. Some studies on parasitic infections have demonstrated that parasites can induce ER stress and subsequent cell apoptosis through the upregulation of GRP78 and caspase 3 expression in infected hosts [59–61]. However, some studies have shown that under damaging pathological conditions, GRP78 has protective effects on tissues or organs *via* Bcl-2 activation [62, 63]. In the central nervous system (CNS), the blood-brain barrier (BBB), which is composed of endothelial cells and astrocytes, is present at blood vessels. This barrier separates the circulating blood from brain tissue and regulates CNS homeostasis. The BBB allows the diffusion of only small hydrophobic molecules (O_2_, CO_2_ and hormones) [64]. Several studies on parasites have demonstrated that pathogens such as *Toxoplasma gondii*, *Toxocara canis*, *Trypanosoma brucei spp.* and malaria can penetrate into the CNS by causing BBB dysfunction [65–67]. Some studies on *A. cantonensis* have found that matrix metalloproteinase induces BBB breakdown and inflammation in cerebral angiostrongyliasis [68, 69]. In this study, we also found that the ESPs of *A. cantonensis* L5 induced the expression of GRP78 and downstream ER stress pathways, including the IRE1, PERK and ATF6 pathways in mouse astrocytes.

Finally, some studies have shown that the Shh pathway has a protective effect on the BBB. Astrocyte-derived Shh proteins can upregulate BBB formation through the stimulation of tight junction protein expression and inhibit proinflammatory cell entry [70–72]. Moreover, Shh signalling protects neurons by inhibiting cell apoptosis in oxidative stress and brain injury. Shh signalling can elevate the expression of antioxidants and anti-apoptotic proteins, including superoxide dismutase, glutathione peroxidase and Bcl-2 [73–77]. In our study, these data indicated that the Shh pathway influenced ER stress by regulating GRP78 expression after treatment with the ESPs of *A. cantonensis* L5 in astrocytes.

## Conclusions

In conclusion, this study found that the ESPs of *A. cantonensis* L5 induce ER stress, upregulate the expression of GRP78 and then activate three ER stress-related pathways, including the IRE1, PERK, and ATF6 pathways. On the other hand, the sonic hedgehog signalling pathway plays an important role in protecting astrocytes by increasing GRP78 expression.

## Data Availability

The data supporting the conclusions of this article are included within the article.

## Abbreviations

L5: fifth-stage larvae
ESPs: excretory/secretory products
ER: endoplasmic reticulum
Shh: Sonic hedgehog
L3: third-stage larvae
UPR: unfolded protein response
GRP78: glucose-regulated protein 78
BiP: binding immunoglobulin protein
Dhh: Desert hedgehog
Ihh: Indian hedgehog
Ptch: Patched
Smo: Smoothened
Gli: Glioma-associated oncogene
FBS: fetal bovine serum
KPBS: potassium phosphate-buffered saline
PFA: paraformaldehyde
CNS: central nervous system
GFAP: Glial fibrillary acidic protein
IHC: immunohistochemical
SAG: Smoothened agonist
r-Shh: recombinant Shh
L1: first-stage larvae
